# The value of lung ultrasound score in neonatal respiratory distress syndrome: a prospective diagnostic cohort study

**DOI:** 10.3389/fmed.2024.1357944

**Published:** 2024-02-08

**Authors:** Chunyan Huang, Shaoqin Zhang, Xiaoming Ha, Yanfang Cui, Hongxia Zhang

**Affiliations:** ^1^Department of Ultrasound, Yantaishan Hospital, Yantai, China; ^2^Medical Impact and Nuclear Medicine Program, Binzhou Medical University, Yantai, China

**Keywords:** neonatal respiratory distress syndrome, lung ultrasound, severity, cohort study, sequential organ failure assessment

## Abstract

**Rationale:**

The accurate diagnosis of critically ill patients with respiratory failure can be achieved through lung ultrasound (LUS) score. Considering its characteristics, it is speculated that this technique might also be useful for patients with neonatal respiratory distress syndrome (NRDS). Thus, there is a need for precise imaging tools to monitor such patients.

**Objectives:**

This double-blind randomized cohort study aims to investigate the impact of LUS and related scores on the severity of NRDS patients.

**Methods:**

This study was conducted as a prospective double-blind randomized study. Bivariate correlation analysis was conducted to investigate the relationship between LUS score and Oxygenation Index (OI), Respiratory Index (RI), and Sequential Organ Failure Assessment (SOFA) score. Spearman’s correlation coefficient was used to generate correlation heat maps, elucidating the associations between LUS and respective parameters in different cohorts. Receiver Operating Characteristic (ROC) curves were employed to calculate the predictive values, sensitivity, and specificity of different scores in determining the severity of NRDS.

**Results:**

This study ultimately included 134 patients admitted to the intensive care unit (ICU) between December 2020 and June 2022. Among these patients, 72 were included in the NRDS cohort, while 62 were included in the Non-NRDS (N-NRDS) cohort. There were significant differences in the mean LUS scores between NRDS and N-NRDS patients (*p* < 0.01). The LUS score was significantly negatively correlated with the OI (*p* < 0.01), while it was significantly positively correlated with the RI and SOFA scores (*p* < 0.01). The correlation heatmap revealed the highest positive correlation coefficient between LUS and RI (0.82), while the highest negative correlation coefficient was observed between LUS and OI (−0.8). ROC curves for different scores demonstrated that LUS score had the highest area under the curve (0.91, 95% CI: 0.84–0.98) in predicting the severity of patients’ conditions. The combination of LUS and other scores can more accurately predict the severity of NRDS patients, with the highest AUC value of 0.93, significantly higher than using a single indicator alone (*p* < 0.01).

**Conclusion:**

Our double-blind randomized cohort study demonstrates that LUS, RI, OI, and SOFA scores can effectively monitor the lung ventilation and function in NRDS. Moreover, these parameters and their combination have significant predictive value in evaluating the severity and prognosis of NRDS patients. Therefore, these results provide crucial insights for future research endeavors.

## 1 Introduction

NRDS is a disease caused by direct or indirect lung injury leading to lung inflammation, significantly affecting the respiratory system and jeopardizing the lives of affected infants (1, 2). It can occur during intrauterine development, the birthing process, and after birth. Thus far, the mortality rate of NRDS remains above 30%, emphasizing the urgent need for timely and effective diagnosis and treatment to prevent infant mortality (3).

In terms of lung examination, lung ultrasound plays a crucial role in swiftly, simply, non-invasively, and radiographically investigating lung lesions in clinical settings (4). Under normal circumstances, the pleura appears as linear hyperechoic lines on ultrasound. Through dynamic observation, respiratory movements cause these pleural lines to slide back and forth, generating horizontal A-lines parallel to the pleural line (5–7). However, when lung tissue undergoes pathological changes, such as the formation of inflammatory exudates and residual gas below the pleura, a perpendicular B-line artifact appears upon ultrasound beam interception (8). The presence of B-lines signifies impaired lung tissue, reduced air content, and increased water content in the respective area (9, 10).

Currently, lung ultrasound focuses primarily on qualitative assessments of neonatal lung conditions, with limited studies exploring quantitative evaluation of changes in lung ventilation areas. However, especially when employing quantitative methods and calculating LUS score, LUS can accurately evaluate lung aeration and facilitate daily diagnosis and monitoring of respiratory issues in critically ill patients. Klaudiusz previously reported the assessment of NRDS severity based on B-lines. Deng et al. (11) research indicated the potential application of LUS scores in evaluating changes in the area of consolidations with re-aeration. Subsequent studies further confirmed the capability of LUS scores in assessing alterations in lung ventilation (12, 13). In a study by Soummer et al. (14), LUS scores were employed to evaluate the occurrence of respiratory distress following a reduction in off-ventilator lung ventilation.

Hence, lung ultrasound holds promising potential in guiding clinical decision-making with regards to respiratory support strategies for NRDS patients. This study aims to utilize LUS as a predictive tool for assessing the severity and prognosis of NRDS, and to evaluate its correlation with traditional parameters, including Oxygenation Index (OI), Respiratory Index (RI), and Sequential Organ Failure Assessment (SOFA) scores.

## 2 Methods

### 2.1 Study design

This study describes a prospective double-blind cohort conducted in a specialized neonatal ICU with an academic focus. The hypothesis tested in this cohort assumes the utility of LUS scoring in predicting the severity and adverse outcomes of NRDS patients. Ethical approval from the local ethics committee as well as written or verbal consent from the parents were obtained upon admission to the neonatal ICU, in accordance with local regulations. The study was conducted following the best practices in prenatal care and international guidelines for resuscitation and respiratory management. The participation in this study did not alter the clinical management, which was provided based on the protocols of the local neonatal ICU. In addition, the STROBE checklist was used to draft this manuscript.

### 2.2 Patients

Within 12 h of admission, all NRDS patients underwent pulmonary ultrasound and other routine examinations. Heart rate, blood gas parameters, and clinical signs were monitored for all patients. Arterial blood samples were collected for blood gas analysis prior to administering any oxygen support. The diagnosis was performed using the Philips CX50, a portable ultrasound device equipped with a linear array transducer operating at a frequency range of 8–12 MHz. The transducer was placed vertically along the intercostal spaces, scanning the patient in a supine, lateral, or prone position, and acquiring transverse and longitudinal images from top to bottom and from left to right, while ensuring the patient remained in a quiet state. Each region was scored based on the presence of the most severe sonographic findings. All examination information is evaluated uniformly by senior sonographers, and the sonograms of each region are stored in real time.

Moreover, the acquisition of clear lung ultrasound images validated the patients’ eligibility for the study. Caregivers of the children were adequately informed about the research and demonstrated their willingness to be part of it. Meanwhile, specific exclusion criteria were established to ensure the integrity of the data analysis. Patients aged above 28 days, with congenital heart or lung disease, or suffering from cardiogenic lung edema were deemed ineligible for enrollment. Any patients with incomplete clinical data that could have potentially influenced the diagnosis or statistical outcomes were also excluded (15, 16).

### 2.3 Data collection

All data were prospectively collected from electronic databases, ensuring the safety of information, and not utilized for routine patient care until the conclusion of the study. In order to ensure the accuracy of lung data in the infants, all participants underwent specialized lung ultrasound performed by trained physicians in the field of ultrasound. One physician was specifically responsible for collecting clinical data and clinical scoring, while another observer performed lung ultrasound and LUS assessments, as well as echocardiography. The blinding of the two observers was maintained throughout the study. All patient-related data remained completely anonymous, and the privacy of the participants was duly respected, with local investigators maintaining identity records. A comprehensive list of the data used and its definitions can be obtained in the study protocol.

### 2.4 Observation and evaluation indicator

**Figure 1:**
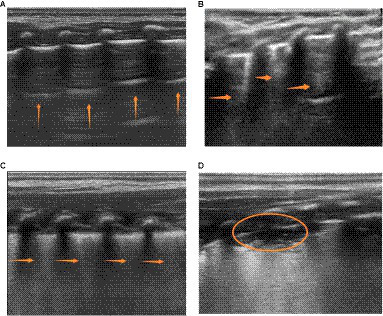
Illustrates the four characteristic patterns of lung ultrasound scores. **(A)** A score of 0 is defined as the presence of only A-lines or < 3 B-lines. A-lines are indicated by vertical arrows. **(B)** A score of 1 is defined as the presence of ≥3 well-spaced B-lines. B-lines are indicated by horizontal arrows. **(C)** A score of 2 is defined as the presence of crowded and coalescent B-lines, with or without subpleural consolidations. **(D)** A score of 3 indicates the absence of spread-pattern consolidations and extensive consolidations with air bronchograms (represented by ellipses).

**Figure 2:**
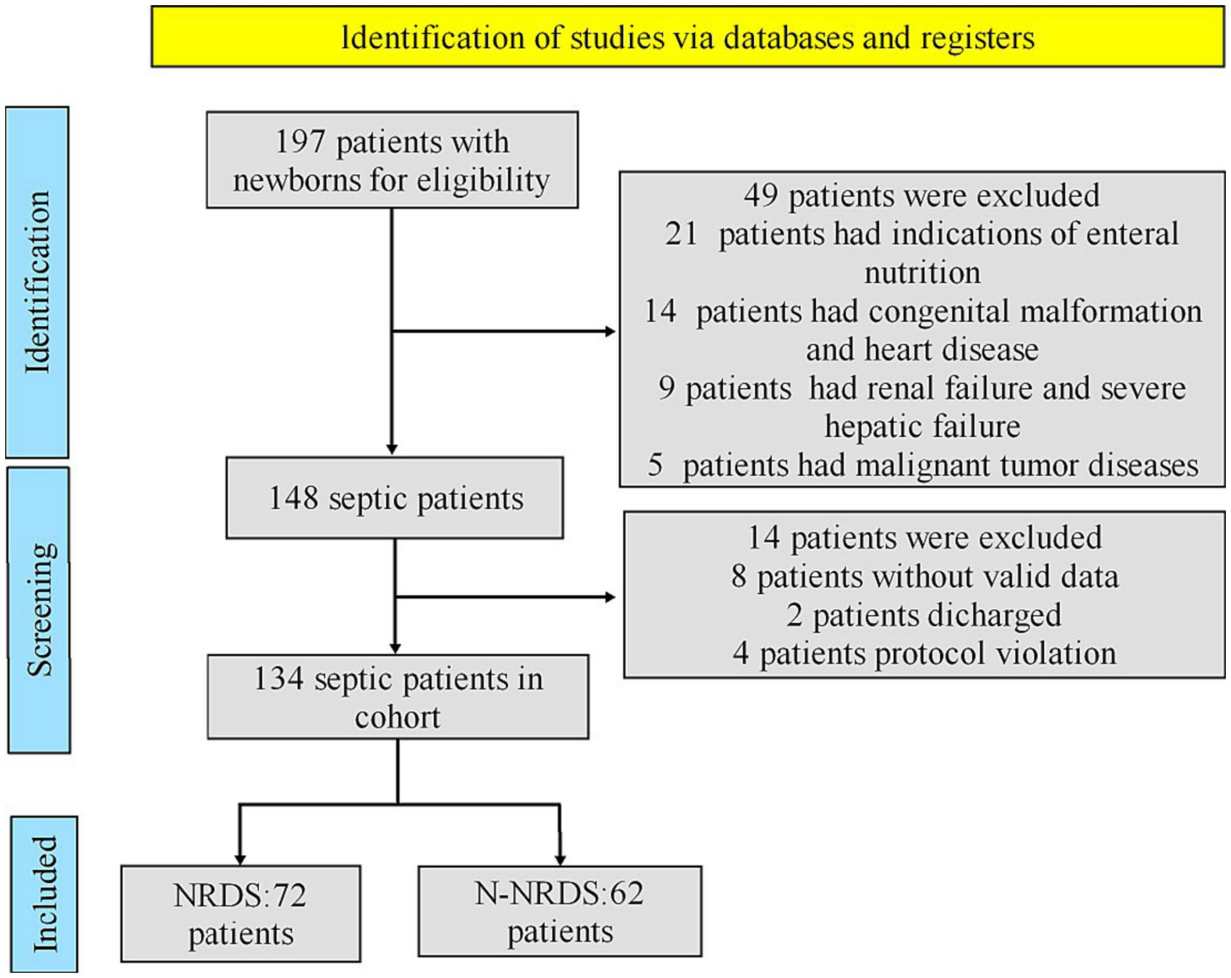
Flow diagram of the study cohort.

1. As indicated in Figure 1, the application of ultrasound examination for evaluating lung areas enables the subdivision of pediatric patients into distinct regions, facilitating a comprehensive assessment of lung conditions. In this study, the ultrasound examination categorized pediatric patients into 12 regions, employing the 12-region scoring method. Taking into account the clinical context, the final LUS is determined by summing up the scores obtained from all 12 regions (17). The scoring system presented in Table 1 offers a standardized approach for quantitatively evaluating the presence and severity of lung abnormalities. A smoothly continuous A-line or less than three isolated B-lines corresponds to a score of 0. Dispersed and clear B-lines are assigned a score of 1. B-lines extensively merging (resembling a waterfall) correspond to a score of 2, while consolidation corresponds to a score of 3. The final LUS score ranges from 0 to 36, as shown in the actual images depicted in Figure 2.
2. The OI calculated as the ratio of arterial oxygen tension (PaO_2_) to inspired oxygen concentration (FiO_2_), typically ranges from 400 to 500 mmHg (18, 19). Clinically, OI is commonly used to assess the oxygenation status of pediatric patients, providing insights into lung gas exchange, ventilation function, hypoxia severity, and lung injury. It also helps gage changes in lung condition and the effectiveness of ventilator therapy. An OI value below 300 mmHg suggests respiratory dysfunction.
3. The RI is calculated as the ratio of the difference between alveolar-arterial oxygen tension and arterial oxygen tension to arterial oxygen tension (20). An RI value greater than 1 generally indicates a significant decrease in oxygenation. Tracheal intubation and mechanical ventilation with supplemental oxygen are recommended when RI exceeds 2 to prevent hypoxia or nitrogen dioxide retention.
4. The SOFA scoring system is a commonly employed tool for evaluating organ dysfunction in pediatric patients (21, 22). It encompasses the respiratory, cardiovascular, neurological, coagulation, hepatic, and renal systems. This scoring system plays a crucial role in predicting the prognosis of pediatric patients.

**Table 1:** Lung ultrasound scoring standard.

Lung ultrasound performance	Score
Smooth A-line or less than 3 isolated B- lines	0
Scattered in clear B-line	1
Numerous fused B-lines (waterfall sign)	2
lung consolidation	3

### 2.5 Outcomes

The primary endpoints of the study include: (1) the correlation of LUS scores with OI, RI, and SOFA scores; (2) the use of LUS, OI, RI, and SOFA to predict NRDS severity. The secondary endpoint is: LUS as the best method for predicting NRDS. The diagnosis of NRDS is typically made by attending clinicians in the absence of lung ultrasound data, as these data are usually only recorded in databases designed for researchers’ use.

### 2.6 Calculations and statistics

Due to the challenges in parameter selection for logistic regression power analysis and retrospective study design, our sample size was calculated with the aim of achieving 80% power at a 0.05 significance level. The sample size calculation was based on the formula: (10*[k + 1]), where k represents the number of explanatory variables in the predictive model.

The datasets in this study were analyzed using SPSS 21.0 statistical software (23, 24). The study employed normally distributed quantitative data expressed as mean ± standard deviation ( $\bar{x}$ ± s). Independent samples t-test was performed to compare two groups, and one-way analysis of variance was employed for comparisons among multiple groups. Pairwise comparisons within groups were conducted using the least significant difference method. The Chi-square test was used to compare count data. Furthermore, a bivariate Pearson correlation analysis was conducted to investigate the correlation between LUS scores and the scores of OI, RI, and SOFA. The ROC curve analysis was used to evaluate the predictive value of LUS, OI, RI, and SOFA in NRDS patients. Kaplan–Meier survival analysis is widely used to evaluate the accuracy of model predictions. Moreover, statistical significance was defined as *p* < 0.05.

## 3 Results

As shown in Figure 2, this cohort study involved a total of 197 patients who stayed in ICU at Yantaishan Hospital between December 2020 and June 2022. Strict inclusion criteria were applied to enroll only those patients meeting the diagnostic criteria for NRDS (25). In a comprehensive evaluation, 63 patients were excluded due to reasons such as indeterminate diagnosis, treatment abandonment, congenital malformation, coexisting tuberculosis or lung cancer, and congestive heart failure. Ultimately, 134 patients were enrolled in the cohort study, with 72 assigned to the NRDS cohort and the remaining 62 to the N-NRDS cohort.

Definition of abbreviations: SGA are small for gestational age. Data are expressed as mean (SD), median (25th percentile–75th percentile), or number (%). *p*-values are calculated with *x*^2^ or Fisher and Student’s or Mann–Whitney test, as appropriate.

The baseline characteristics of the two cohorts are presented in Table 2. In the NRDS cohort, the male proportion was 52.8%, with a gestational age of (29 ± 3.2) weeks and a weight of (1,865 ± 571) grams. Cesarean section accounted for 65.3% of deliveries, while 40.3% of the patients required invasive ventilation within 24 h.

**Table 2:** Primary outcome.

Name	Whole population (134)	NRDS Cohort (72)	N-NRDS Cohort (62)	*p*-value
Gestational age, wk	30 ± 3.1	29 ± 3.2	32 ± 2.9	<0.01
Birth weight, g	2,078 ± 621	1,865 ± 571	2,254 ± 726	<0.01
SGA neonates	41 (30.6%)	27 (37.5%)	14 (22.58%)	0.09
Sex, M	71 (53%)	38 (52.8%)	33 (53.2%)	0.412
Cesarean section	83 (61.9%)	47 (65.3%)	36 (58%)	<0.01
Antenatal steroids	108 (81%)	59 (82%)	49 (79%)	0.153
Needing invasive ventilation for: 24 h	37 (27.6%)	29 (40.3%)	8 (12.9%)	<0.01

### 3.1 Comparison of clinical indicators in patients with different NRDS conditions

The LUS score for NRDS group infants was 21.88 ± 6.87, while the LUS score of infants in the N-NRDS group was 15.96 ± 3.51. The OI for NRDS group infants was 179 ± 75, while the OI for N-NRDS group infants was 243.3 ± 76.3. Additionally, the RI for NRDS group infants was 1.65 ± 0.56, in contrast to the LUS score of 1.15 ± 0.41 for N-NRDS group infants. Moreover, the SOFA score for NRDS group infants was 21.88 ± 6.87, while the N-NRDS group was 15.96 ± 3.51. The intergroup differences in these various parameters were all statistically significant (*p* < 0.01) (Table 3). Notably, the N-NRDS group infants exhibited significantly lower LUS, RI, and SOFA scores compared to the NRDS group, indicating a more desirable outcome. However, their OI values were higher than those observed in the NRDS group, suggesting a differing response to treatment (p < 0.01).

**Table 3:** Presents a comparison of the LUS scores and lung function indicators between the two groups of patients.

	Whole population	NRDS	N-NRDS	*p*-value
LUS	19.77 ± 4.83	21.88 ± 6.87	15.96 ± 3.51	<0.01
RI	1.33 ± 0.5	1.65 ± 0.56	1.15 ± 0.41	<0.01
OI	205.8 ± 79.3	179.2 ± 75	243.3 ± 76.3	<0.01
SOFA	15.31 ± 4.21	16.17 ± 4.8	13.65 ± 3.9	<0.01

NRDS, neonatal respiratory distress syndrome; N-NRDS, Non-Neonatal respiratory distress syndrome; LUS, lung ultrasound; RI, respiratory index; OI, oxygenation index; SOFA, sequential organ failure assessment.

### 3.2 The relationship between LUS and RI, OI, and SOFA in NRDS

**Figure 3:**
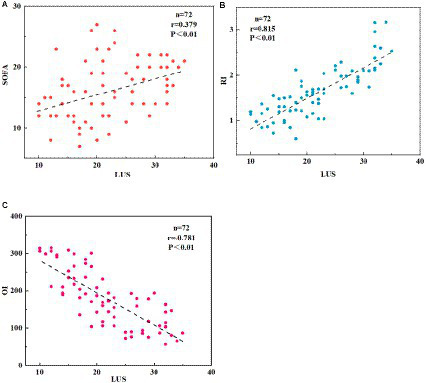
Displays the correlation between LUS, RI, OI, and SOFA scores in NRDS patients. **(A)** The correlation between LUS and SOFA scores in NRDS. **(B)** The correlation between LUS and RI scores in NRDS. **(C)** The correlation between LUS and OI scores in NRDS. NRDS refers to neonatal respiratory distress syndrome, LUS represents lung ultrasound score, RI denotes respiratory index, OI represents oxygenation index, and SOFA stands for sequential organ failure assessment.

The correlation between LUS scoring and RI, OI, and SOFA scoring was analyzed using Pearson correlation analysis, as depicted in Figure 3. In the NRDS group, as the severity of the patients’ condition worsened, their LUS, RI, and SOFA indices increased, whereas the OI index decreased. The LUS score was significantly negatively correlated with the OI (*p* < 0.01), while it was significantly positively correlated with the RI and SOFA scores (*p* < 0.01). The LUS score was positively correlated with the SOFA score (*r* = 0.379, *p* < 0.01), indicating that as the LUS and SOFA scores increase, the condition of NRDS patients becomes more severe. Furthermore, it is worth noting that the correlation between LUS and RI is stronger compared to the correlation between LUS and SOFA.

### 3.3 Correlations between LUS and lung parameters in different cohorts

**Figure 4:**
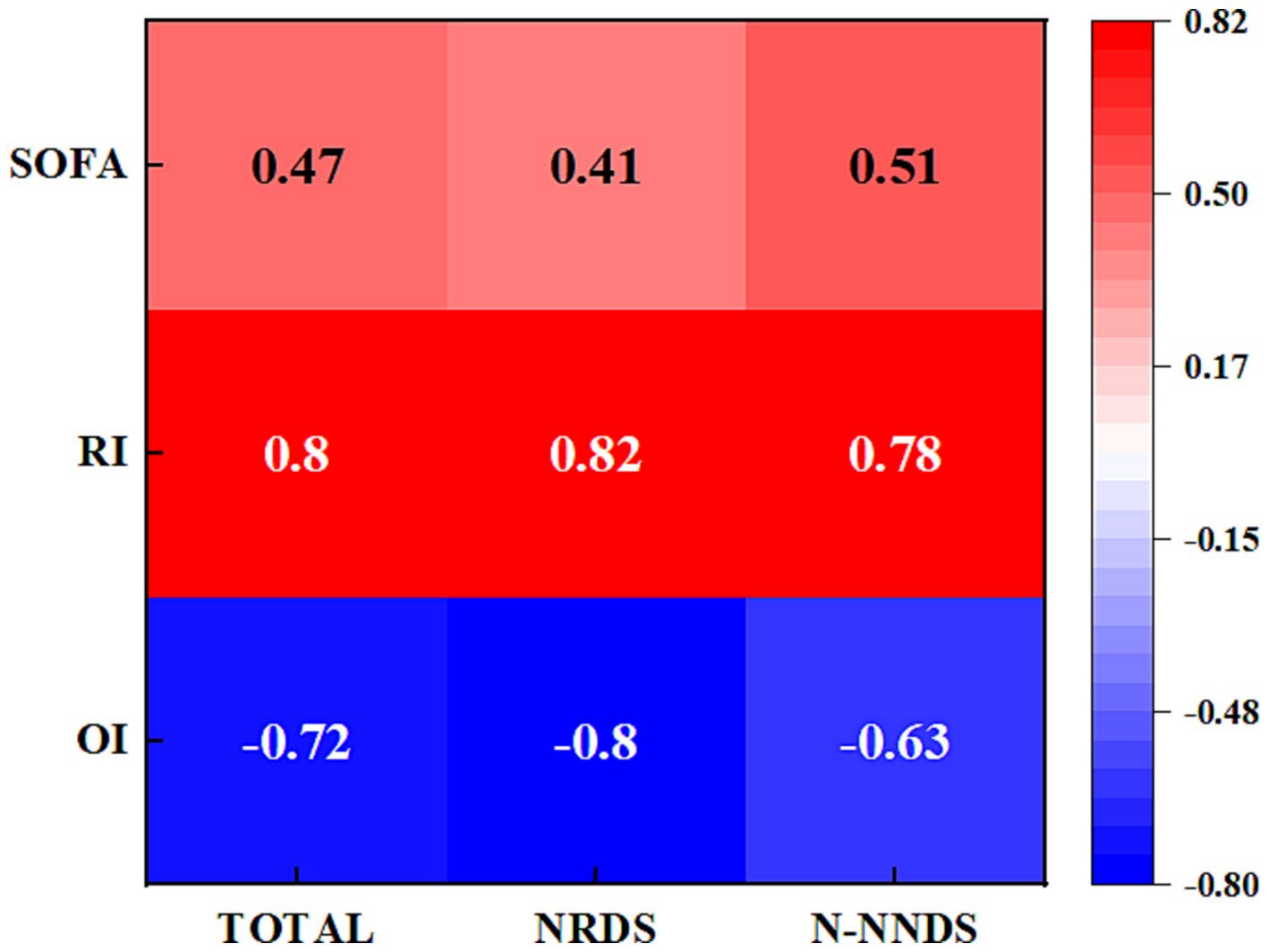
Displays the heatmap illustrating the correlations between LUS and the relevant parameters in different cohorts. The numeric values represent the Spearman correlation coefficients, ranging from −0.8 to 0.82.

Figure 4 illustrates the correlation heat map between LUS and RI, OI, and SOFA in different cohorts. The study results indicate an association between LUS and other scores in the entire patient population as well as in NRDS and non-NRDS cohorts. LUS is positively correlated with RI and SOFA, and negatively correlated with OI (*p* < 0.01). This suggests that LUS can serve as an important indicator for assessing the pulmonary pathology and disease severity in these patients. In the NRDS cohort, the positive correlation between LUS and RI is particularly significant (*p* < 0.01). Specifically, in the NRDS cohort, the highest positive correlation coefficient observed was 0.82. This indicates a close relationship between pulmonary ultrasound imaging features and RI in NRDS patients, possibly due to more severe respiratory issues in NRDS patients. Furthermore, in the NRDS cohort, the negative correlation between LUS and OI is also significant (−0.8). This suggests that an increase in LUS is associated with a decrease in OI in these patients.

### 3.4 The value of LUS combined with other scores in predicting the severity of NRDS

The prognostic value of LUS combined with lung related indicators was investigated in NRDS patients. Table 4 shows the predicted results for NRDS. Research has found that LUS has a higher AUC value in predicting the severity of NRDS patients compared to RI, OI, and SOFA scores. Moreover, the accuracy of LUS is significantly higher than that of each individual indicator (*p* < 0.01).

**Table 4:** Diagnostic value of NRDS with different scores.

Name	Cut-off	Sensitivity (%)	Specificity (%)	AUC	95% CI	Youden index	*p*-value
LUS	20.5	88.1	83.1	0.91	0.84–0.98	0.77	<0.01
RI	1.57	86.7	82.2	0.88	0.81–0.95	0.71	<0.01
OI	181	82.7	81.9	0.86	0.79–0.93	0.68	<0.01
SOFA	14.5	80.5	80	0.75	0.77–0.83	0.57	<0.01
Combination	–	92.2	83.3	0.93	0.85–0.98	0.81	<0.01

**Figure 5:**
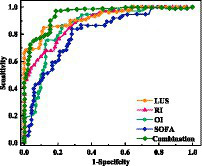
Diagnostic value of LUS, RI, OI, and SOFA score in NRDS.

Furthermore, ROC curves were constructed using NRDS as the study population. By analyzing the ROC curves (Figure 5), it was found that when the LUS score exceeds 20.5, it suggests a critical condition in pediatric patients, with a sensitivity of 88.1% and specificity of 83.1%. The AUC is 0.91 (95%CI: 0.84–0.98). When the RI is above 1.57, it indicates an extremely critical condition, with a sensitivity of 86.7% and specificity of 82.2%. The AUC is 0.88 (95% CI:0.81–0.95). OI below 181 suggests a critical condition, with a sensitivity of 82.7% and specificity of 81.9%. The AUC is 0.86 (95% CI:0.79–0.93). Finally, a SOFA score above 14.5 indicates a critical condition, with a sensitivity of 80.5% and specificity of 80%. The AUC is 0.75 (95% CI:0.77–0.83). It is noteworthy that the combination of LUS with other scores can more accurately predict the severity in NRDS patients, with the highest AUC value of 0.93, which is significantly higher than using individual indicators alone (*p* < 0.01), with the highest sensitivity and specificity reaching 92.2 and 83.3%, respectively.

## 4 Discussion

NRDS is a very serious lung disease, and timely and accurate assessment of the patient’s condition is of great significance for clinical treatment (26). Lung ultrasound has the advantages of simplicity, radiation-free imaging, and excellent repeatability. In a normal lung, the pleural lines appear smooth or exhibit multiple parallel high echogenicity lines, while severe pneumonia is characterized by changes in the gas–liquid ratio, lung tissue aeration, and consolidation (27–29). LUS enables the quantification of the patient’s condition, accurately reflecting the severity of the illness. The RI represents the ratio of alveolar-to-arterial oxygen tension difference (30). An RI greater than 1 is indicative of significantly reduced oxygenation, while an RI above 2 necessitates the use of endotracheal intubation and mechanical ventilation with oxygen for avoiding hypoxia and nitric oxide retention (27). An OI in the normal range of 400–500 mm Hg reflects the degree of hypoxia, with lower values indicating impaired lung gas exchange (31, 32). SOFA scores assess the respiratory, coagulation, liver, circulatory, neurological, and renal systems, thereby providing an overview of the patient’s overall organ function. Higher SOFA scores correspond to more severe illness (33). This study aims to explore the correlation between LUS and the severity and prognosis of NRDS. The results indicate significant differences (*p* < 0.01) in LUS, RI, OI, and SOFA scores among different patient cohorts, emphasizing the close association of these indicators with NRDS severity. Furthermore, the LUS, RI, and SOFA scores were higher in the NRDS cohort compared to the N-NRDS cohort, whereas the OI was lower. This suggests that as the illness progresses, LUS, RI, and SOFA scores increase, while the OI decreases. Therefore, the value of these four indicators in assessing the severity of NRDS is evident. These findings are in line with the conclusion drawn by Yue et al. (34). Ultrasound waves encounter significant acoustic impedance and speed variations across different media (35, 36). The coexistence of fluid and gas in lung tissue leads to complete reflection, resulting in artifacts such as B-lines or A-lines. Noticeable changes in the fluid-to-gas ratio across different lung tissue types represent varying degrees of lung tissue aeration loss. Severe involvement results in contiguous and fused B-lines, while moderate involvement is characterized by multiple well defined B-lines (37). Pearson correlation analysis revealed a negative correlation between LUS and OI, and positive correlations between LUS and RI, OI, and SOFA scores (all *p* < 0.01), indicating that LUS accurately reflects NRDS progression. This finding is consistent with the research conducted by Senter et al. (38), supporting the use of LUS as an auxiliary tool for evaluating the effectiveness of NRDS treatment.

The findings of this study underscore the importance of LUS and other indices in predicting the severity of illness in NRDS patients (39, 40). The accuracy of predicting the severity of NRDS patients can be enhanced through the combined use of LUS, RI, OI, and SOFA scores. Analysis from this research has revealed that for assessing the severity of NRDS, an LUS ≥ 20.5, RI ≥ 1.57, OI ≥ 181, and SOFA≥14.5 correspond with an AUC greater than 0.75, exhibiting high sensitivity and specificity. These findings suggest that scoring indicators such as LUS can effectively predict the severity of illness in NRDS patients. Moreover, the AUC for the combined prediction of the severity of NRDS utilizing all four indicators surpasses each individual marker, implicating that their integrated application enhances predictive accuracy. This result provides a significant clue that by evaluating markers such as LUS, RI, OI, and SOFA, we can more precisely ascertain the condition and disease severity in NRDS patients (41–43). Previous studies have also identified LUS scoring as a safe, cost-effective, and straightforward tool, which is easily utilized and accessed, embodying substantial practical value. Zhang et al. (44) also discovered that when the LUS score reached 19.50, it showed good predictive value regarding the severity and prognosis of NRDS, serving as a reliable prognostic marker. Additionally, literature reports have highlighted that LUS levels were significantly higher in the non-survivor group compared to the survivor group, positively correlating with disease severity (45, 46). Therefore, it is one of the valuable indicators for assessing the severity and prognosis of NRDS patients. This holds considerable value to clinicians, as accurately determining disease severity can guide clinical treatment and prognostic evaluation, contributing to enhanced patient survival rates and outcomes.

## 5 Conclusion

Our prospective double-blind randomized controlled trial demonstrates that LUS, RI, OI, and SOFA scores can effectively monitor the lung ventilation and function in NRDS. Moreover, these parameters and their combination have significant predictive value in evaluating the severity and prognosis of NRDS patients. Consequently, these findings can be utilized to characterize the features and individualize respiratory care for patients, as well as explore novel therapeutic interventions.

## Data availability statement

The original contributions presented in the study are included in the article/supplementary material, further inquiries can be directed to the corresponding author.

## Ethics statement

This observational and retrospective study was approved by the Clinical Ethics Committee of Yantaishan Hospital, Shandong Province, China (Ethical Review No. 20220001), and informed consent was obtained from each child’s guardian. The studies were conducted in accordance with the local legislation and institutional requirements. Written informed consent for participation in this study was provided by the participants’ legal guardians/next of kin. Written informed consent was obtained from the individual(s), and minor(s)’ legal guardian/next of kin, for the publication of any potentially identifiable images or data included in this article.

## Author contributions

CH: Methodology, Project administration, Resources, Software, Writing – original draft. SZ: Formal analysis, Investigation, Writing – review & editing. XH: Funding acquisition, Investigation, Methodology, Writing – original draft. YC: Data curation, Writing – review & editing. HZ: Funding acquisition, Writing – original draft.
